# Effect of Laser Photobiomodulation on Postoperative Pain After Single-Visit Endodontic Treatment in Children: A Randomized Control Trial

**DOI:** 10.3390/children11121511

**Published:** 2024-12-12

**Authors:** Yashaswini S. Angolkar, Sadanand Kulkarni, Chandrashekar M. Yavagal, Puja C. Yavagal, Umesh Bhosle, Viplavi Chavan Patil, Sultan Abdulrahman Almalki, Inderjit Murugendrappa Gowdar, Khalid Gufran

**Affiliations:** 1 Department of Pediatric Dentistry, Maratha Mandal’s Nathajirao G. Halgekar Institute of Dental Sciences & Research Centre, Belgaum 590019, India; drsadanandkulkarni@hotmail.com (S.K.); umeshmbhosle93@gmail.com (U.B.); viplavi44@gmail.com (V.C.P.); 2 Department of Pedodontics and Preventive Dentistry, Bapuji Dental College and Hospital, Davanagere 577004, India; dryavagal@gmail.com; 3 Department of Public Health Dentistry, Bapuji Dental College and Hospital, Davanagere 577004, India; pujacyavagal@gmail.com; 4 Department of Preventive Dental Sciences, College of Dentistry, Prince Sattam bin Abdulaziz University, Alkharj 11942, Saudi Arabia; s.almalki@psau.edu.sa (S.A.A.); i.gowdar@psau.edu.sa (I.M.G.); k.syed@psau.edu.sa (K.G.)

**Keywords:** children, endodontic pain, laser, photobiomodulation, pulpectomy

## Abstract

Background: This study aimed to assess the effectiveness of laser photobiomodulation (PBM) in reducing postoperative pain following single-visit endodontic treatment in children aged 5–9 years. Methods: Forty children aged 5–9 years with acute irreversible pulpitis in deciduous molars requiring single-visit pulpectomy were included in the study. Pulpectomy was performed according to a standard endodontic protocol. The participants were randomly allocated to two groups: group A received laser photobiomodulation using an 810 nm diode laser applied to the periapical area of the treated tooth postoperatively, and group B received blue light LED, applied at similar points to the control teeth. This was a placebo intervention. Pain scores were measured using a 10-point Visual Analog Scale (VAS) at 4, 12, 24, and 48 h post-treatment. The Friedman test was used to compare the VAS scores within groups over time, and the Mann–Whitney U-test was used to compare the VAS scores between the two groups. The significance level was fixed at *p* < 0.05. Result: The mean VAS scores were significantly lower in the PBM group compared to the placebo group at the 4th h, 12th h, and 24th h post-treatment (*p* ≤ 0.05). The pain levels remained stable over time in the PBM group, with no significant difference in pain scores from 4 h to 48 h (*p* = 0.57). In the placebo group, the pain gradually decreased from the 4th h to the 48th h, with a significant reduction in pain observed between the 12th h and 48th h (*p* = 0.05). Conclusions: Laser photobiomodulation (PBM) was found to be effective in reducing postoperative pain following single-visit endodontic treatment in children. This non-invasive approach could offer a valuable alternative for pain management in pediatric endodontics, particularly given its effectiveness without the need for systemic medications.

## 1. Introduction

Acute irreversible pulpitis is a common condition in pediatric patients, typically requiring either pulp therapy (such as pulpectomy) or extraction when the tooth cannot be preserved. In these cases, post-treatment pain and inflammation are common postoperative complications, despite proper treatment of the pulp and a good coronal seal. Effective and predictable pain management is a cornerstone of pediatric dentistry, especially because children’s responses to pain and treatments are often different from adults. Advances in single-visit endodontics have become a significant focus in pediatric dentistry because they simplify treatment, reduce the need for multiple appointments, and help minimize the stress that children may feel from repeated visits [1,2]. Despite these advancements, postoperative pain and swelling are common side effects following root canal treatments in children. Even though root canals are prepared chemo-mechanically and obturating material is placed with an adequate coronal seal to prevent infection, there can still be tissue irritation, inflammation, or sensitivity, leading to pain [3]. Predictable pain management is a cornerstone of modern healthcare, especially in pediatric dentistry. Pain management is not just about alleviating discomfort; it also plays a critical role in behavior management, as pain can directly influence a child’s cooperation and their overall perception of dental care. Different strategies to manage postoperative pain include non-steroidal anti-inflammatory analgesics, which, however, have their own side effects [3,4]. Photobiomodulation (PBM) therapy has shown significant promise in various clinical applications, including pain relief, wound healing, and nerve damage repair [1,5]. The rapid developments in laser technology have deepened our understanding of biological mechanisms, leading to significant implications and improvements in an individual’s quality of life [6,7]. The work of Tiina Karu in the early 1990s has been pivotal in shedding light on the oxidation–reduction (redox) reactions that are at the core of how laser light interacts with biological tissues [8]. Particularly notable is its effectiveness in endodontic therapy, where it serves to disinfect root canals and reduce pain during procedures [9]. One of the major advantages of PBM is its safety profile, as it has demonstrated effectiveness without causing significant side effects, making it an attractive therapeutic option for dental treatments. The mechanisms underlying PBM’s ability to alleviate pain and promote healing are not fully understood, but there are several proposed theories. A key hypothesis is that PBM may reduce pain by increasing adenosine triphosphate (ATP) production in cells, which helps improve cellular function [10]. Additionally, PBM is believed to decrease oxidative stress in tissues, contributing to its therapeutic effects. A widely accepted mechanism of action involves the absorption of near-infrared light by target tissues, such as those in the oral cavity, during laser therapy. The absorbed light generates reactive oxygen species (ROS) that activate cellular signaling pathways, including gene transcription. This process ultimately promotes cellular healing and tissue repair. Mitochondria, the energy-producing organelles in cells, are particularly responsive to this light-induced process. By converting absorbed light into ATP, PBM enhances cellular energy, supporting various cellular functions that are crucial for tissue regeneration and healing [10]. With its safety, efficacy, and non-invasive nature, PBM is becoming an increasingly popular tool in pediatric dentistry, particularly for managing post-surgical pain, reducing medication use, and improving patient comfort [9]. However, there is a paucity of literature regarding the same in pediatric endodontic procedures. Hence, the purpose of this study was assessing the efficacy of laser PBM in managing postoperative pain after single-visit endodontic treatments in children aged 6–9 years. The study tested the null hypothesis, which states that there would be no significant difference in the Visual Analogue Scores (VAS) of pain between the PBM group and the placebo group at different time points: 4 hours, 12 hours, 24 hours, and 48 hours after treatment.

## 2. Materials and Methods

**Figure 1:**
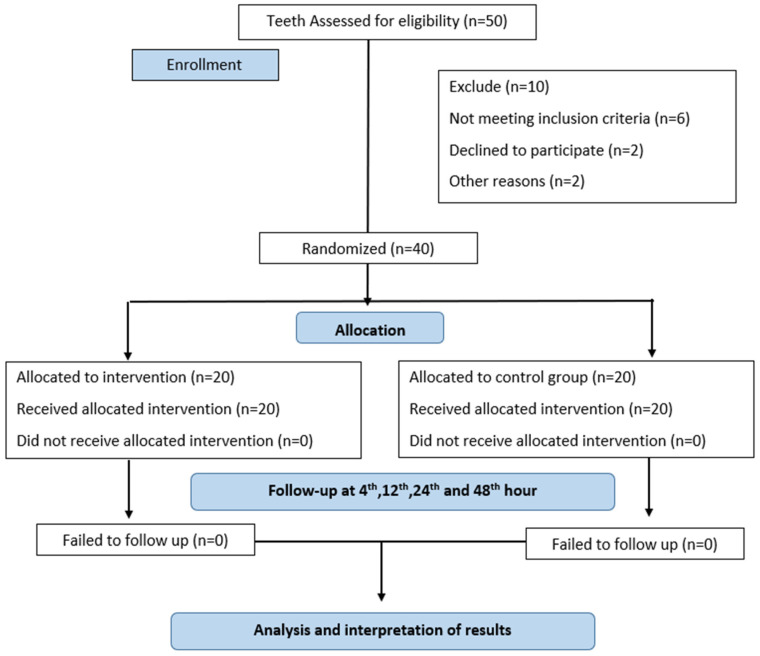
CONSORT flow diagram of study methodology.

Study design was an in vivo, randomized controlled, parallel group, active arm clinical trial. The study methodology is depicted in the flow chart (Figure 1). The study was carried out in the clinics of the Department of Pediatric and Preventive Dentistry, Maratha Mandal’s Nathajirao G. Halgekar Institute of Dental Sciences & Research Centre, Belgaum, India.

The institutional ethics review board of the hospital where the study was carried out granted ethical clearance. (Ref No.: 7-6-2022, 2022-23/364). Voluntary written informed consent and assent was obtained from the parents of the participants before the start of the study and after explaining the procedural details, harms, and benefits of the intervention to the parents using a participant information form. The trial is registered on Thai Clinical Trial Registry (TCTR) with the ID: TCTR20241026005; https://www.thaiclinicaltrials.org/show/TCTR20241026005, accessed on 3 October 2024.

The study involved children aged 5–9 years who met specific criteria for participation. Eligible participants were those who were not on any medications, had teeth identified as needing pulpectomy, had teeth with 2/3 of intact root length, and were diagnosed with irreversible pulpitis without any signs of swelling, periapical abscess, and/or pathological/physiological mobility. The study excluded teeth with radiographic evidence of internal resorption, pulp calcification, and osseous disease. The study employed a consecutive sampling procedure to include only one treated tooth per participant.

The Institute for Experimental Psychology in Dusseldorf, Germany’s G*Power version 3.1.9.7 for Windows XP was used to determine the sample size. The effect size was determined using the averages and standard deviations from a prior study conducted by Cakici et al. [1]. Given the significance level of *p* ≤ 0.05, effect size of 0.9, and study power of 0.8, the anticipated sample size was 40 teeth, with 20 teeth in each group.

Randomization:

The patients were randomly assigned to the interventional groups once the clinical and radiographic characteristics of the chosen tooth had been standardized. The teeth were allocated by a concealed random allocation to the interventional groups. A computer-generated random sequence of code A and code B (total of 40) was used to allocate the teeth to the two interventional groups. The codes were placed in opaque, concealing covers according to the sequence. Another individual who was not participating in the study carried out the random allocation.

Interventional groups:

Group A (Experimental group): Photobiomodulation was perfprmed using 810 nm diode laser (Novolase Pedo PRO, Novolase Technologies, Mumbai, India) postoperatively at the periapical area of endodontically treated tooth, and Group B (Control group): Blue light LED (Placebo group) was applied postoperatively at the periapical area of endodontically treated tooth.

Calibration of examiners:

All treatments were performed by the same researcher (Operator 1). Pain levels were assessed by a separate, previously calibrated examiner (Operator 2). To minimize errors and avoid bias, Operator 2 was blinded to the treatment group of each patient. After assessing the response of each tooth to pain stimuli, Operator 2 measured and recorded the levels of post-endodontic pain using the Visual Analog Scale (VAS).

**Figure 2:**
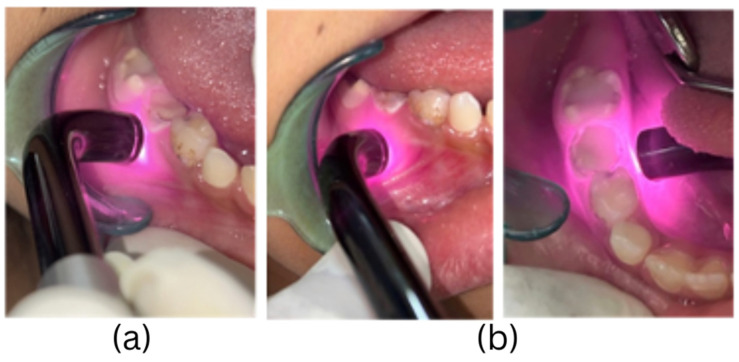
Laser irradiation using 810 nm diode after endodontic therapy at 2 points: (**a**) cervical and (**b**) apical on both buccal and lingual aspects.

Intervention details:

After single-visit endodontic procedure of the selected tooth was carried out under local anesthesia using the standard amount of 2% lignocaine with 1:80,000 adrenaline (Xicaine, ICPA, Ankleshwar, India), the tooth received one of the following interventions:

Experimental group intervention: Laser photobiomodulation was performed at 4 sites on each treated tooth, with 15 s each for the periapex and cervical area of the endodontically treated tooth on both the buccal and lingual sides, giving a total exposure time of 60 s (4 × 15 s) for each tooth. The 810 nm laser was operated in continuous wave mode and was delivered through an 8 mm diameter tip used in contact mode, giving a spot size of 0.5 cm^2^. The power output was 200 mW, and the power density during irradiation was 397 mW/cm^2^. The energy dose was 3 Joules per treated point and, thus, 12 Joules per tooth (Figure 2). PBM was performed immediately after the endodontic treatment and restoration. 

**Figure 3:**
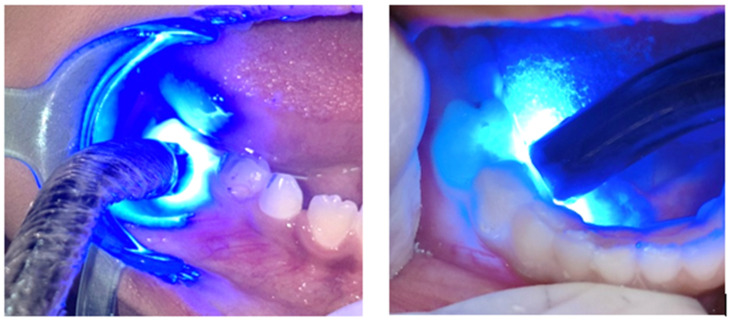
Placebo LED light irradiation.

Control group intervention: A blue LED curing light (Woodpecker Cordless LED-C (Guilin Woodpecker Medical Instrument Co., Guilin, China)) of 420–480 nm wavelength was held at the four tooth sites similar to that of the interventional group for the same exposure time of 15 s per site. The curing light had a tip diameter of 12 mm (spot size 1.13 cm^2^), and this was used in non-contact mode and kept 1 mm away from the tissue (Figure 3). The measured emitted power density was 700 mW/cm^2^. This was not sufficient to cause photothermal heating of soft tissues during the exposure times used. Additionally, subjects were to raise their hands if warmth or discomfort was felt during the LED light exposure. No subjects did this, nor did they report discomfort from the LED control treatment.

Pain Assessment:

The intensity of pain was measured and documented by using 10-point Visual Analogue Scale [1]. Each patient was given a form/chart after the treatment and instructed to mark the score according to the intensity of pain at the 4th, 12th, 24th, and 48th hours after the treatment. An analgesic tablet, containing a combination of Ibuprofen and paracetamol and dosed at 10 mg/kg based on the body weight of the child, was prescribed as a postoperative measure to alleviate pain in the patient (as optional) if the postoperative pain was unbearable, and such a participant’s data were not considered for analysis. Pain was recorded personally by the investigator at all assessment time periods by meeting the child in person at their residence.

Blinding:

Only the statistician was blinded to the group allocation. Participants and investigators were not blinded.

Safety Protocol:

All clinical procedures were carried out under strict aseptic precautions. Participants and investigators wore wavelength-specific eyewear during intervention.

### Statistical Analysis

The statistical analysis was conducted using IBM SPSS Statistics Version 20 for Windows, version 20 (IBM Corp., Armonk, NY, USA). Because the data did not follow a normal distribution, nonparametric tests were employed for data analysis. The significance level was set at *p* < 0.05. The means of VAS scores within and between groups were compared using the Friedman test and the Mann–Whitney U-test, respectively.

## 3. Results

The mean ages of the study participants in the control and PBM groups were 6.32 ± 1.84 and 6.90 ± 1.21, respectively. The control group consisted of 8 male and 12 female children, whereas the PBM group had an equal number of male and female children. The median VAS score for both the control and PBM groups was 4. In the control group, 12 deciduous first molars and 8 deciduous second molars were treated with endodontics, whereas in the PBM group, 13 first molars and 7 second molars received treatment. The baseline characteristics of the two groups were not significantly different, as shown in Table 1.

The study’s findings regarding pain scores, measured by median VAS scores, are summarized in Table 2. The PBM (Laser) group showed significantly lower pain scores compared to the control (LED) group at the 4th, 12th, and 24th h, with a *p*-value ≤ 0.05. By the 48th h, the pain score was 0 in both groups, indicating no pain. In the PBM group, pain levels remained consistent across all time points, with no significant change (*p* = 0.57), suggesting stable pain levels. In the control group, pain gradually decreased from the 4th h to the 48th h, with a significant reduction observed between the 12th and 48th h (*p* = 0.05), indicating a gradual improvement in pain over time. These results suggest that while the PBM group experienced significant pain relief at earlier time points, both groups reached a similar outcome of no pain after the 48th h.

**Figure 4:**
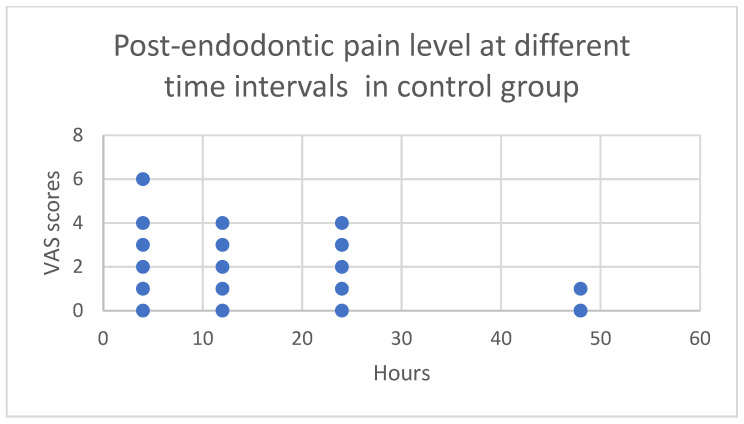
Post-endodontic pain level at different time intervals in control group.

**Figure 5:**
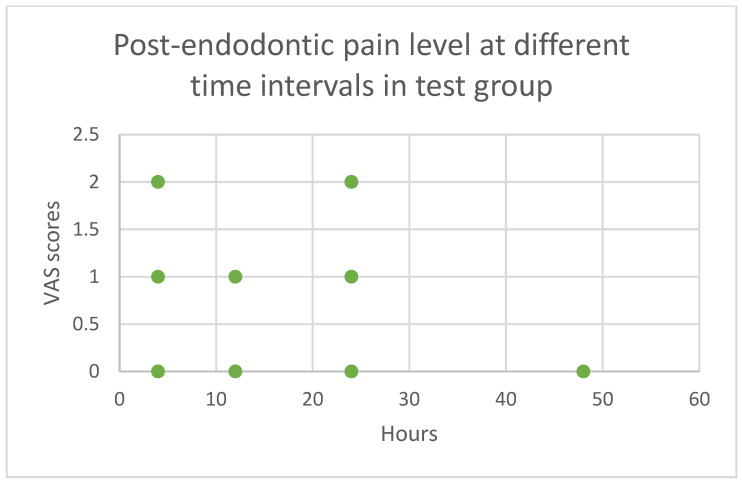
Post-endodontic pain level at different time intervals in test group.

Figure 4 (control group) and Figure 5 (test group) display scatter plots showing the actual data points for the VAS scores at each time point. In the control group, the pain scores ranged from 0 to 6 at the 4th h after post-endodontic treatment. As time progressed, the pain levels gradually decreased, with a median VAS score ranging from 0 to 4 at the 12th and 24th h. By the 48th h, the pain levels had decreased to nearly zero. In the test group, the pain scores remained consistent (ranging from 0 to 2) across all time points and reduced to zero by the 48th h.

## 4. Discussion

The findings of the present study suggest that laser photobiomodulation (PBM) effectively reduced postoperative pain following single-visit endodontic treatment in pediatric patients. These results are consistent with previous studies that have reported similar outcomes, showing that PBM can be beneficial in managing post-endodontic pain after single-visit root canal therapy [3,11,12].

Pain is indeed a multidimensional experience, combining sensory (physical discomfort) and cognitive (emotional and psychological) components. In the context of dental procedures, such as pulpectomy, postoperative pain is a common occurrence [6]. This pain can be problematic not only due to the immediate physical discomfort but also because it can affect the psychological well-being of patients, particularly in pediatric populations [13,14]. Children with acute irreversible pulpitis are at a significantly higher risk of experiencing postoperative pain compared to those with necrotic pulps. Specifically, the risk is 8.72 times greater in children with acute irreversible pulpitis [15]. The increased likelihood of postoperative pain in children with acute irreversible pulpitis highlights the need for effective pain management strategies in pediatric dentistry [15].

Alnassar et al. [16] suggested that the incidence of post-endodontic pain tends to decrease gradually over time after root canal procedures. This is typical, as acute discomfort following the procedure may subside as the tissues heal and inflammation reduces. The decrease in pain over time may also indicate the effectiveness of the treatment in addressing the underlying infection or inflammation. Research has shown that nonvital teeth tend to have a higher incidence of postoperative pain compared to vital teeth. This is likely because nonvital teeth often have more complex issues, such as infection or inflammation of surrounding tissues, which can lead to a more intense inflammatory response after treatment [17]. Martins et al. [18] suggest that extrusion of root canal filling material beyond the root apex (the tip of the root) can lead to complications, including inflammatory cellular elements, elevated fibrous tissue concentration, and micro abscess formation. Effective pain management is a critical aspect of endodontic treatment, playing a vital role in both the immediate experience of the patient and the long-term success of the procedure. Proper pain management involves technical considerations and the use of appropriate solutions to reduce discomfort, prevent complications, and promote healing [19]. The most preferred pharmaceutical remedies for pain management in pediatric dentistry are NSAIDs, which relieve pain by decreasing the chemical inflammatory mediators in peripheral nociceptors, thus triggering the associated subsequent events [19]. In addition, NSAIDs are noted for their side effects; several studies have shown a significant proportion of patients experiencing symptoms of upper GI disorders, associated with hepatotoxicity, increased risk of kidney disorders, etc. [19,20]. Hence, given the wide range of different technical approaches proposed to manage postoperative endodontic pain, it is crucial to consider all options.

Photobiomodulation therapy (PBM) is a promising, non-invasive, and effective method for managing postoperative pain in pediatric dentistry. The ability to modulate biological processes without causing harm to the tissues makes PBM an appealing option, especially in young patients who may have heightened sensitivities to invasive treatments or medications. However, the success of PBM depends on the careful consideration of key parameters such as laser type, wavelength, energy density, and exposure time, all of which must be tailored to the specific needs of the patient and the nature of the dental procedure [5,6,21]. Among the various lasers, diode lasers are the most frequently used. In the current study, the wavelength of the laser unit used was 810 nm (Novolase technologies, India), which conformed to the optimal optical range with a power of 200 mW for 60 s. The diode laser typically uses a solid semiconductor made of aluminum gallium arsenide as the active medium. It operates specifically between 808 and 980 nm. This wavelength is readily absorbed by tissues containing hemoglobin, melanin, and collagen chromospheres, but it shows little absorption by hard dental tissue [22,23].

According to the literature, very short wavelengths below 350 nm are known as ionizing and have the ability to deeply penetrate biological tissue, cleave cellular DNA, and cause mutagenic effects [4].

Wavelengths greater than 350 nm can cause excitation and heating of the tissue with which they interact but cannot cleave their DNA. PBM has regenerative effects, reduces C fiber activity, and prevents the production of inflammatory substances [4,22]. Thus, the 810 mm wavelength in continuous mode exhibits selective action and is recommended for inhibiting inflammatory processes and promoting tissue healing in the current study. In the continuous-wave mode, light is emitted continuously at a consistent power level. The target tissue continuously absorbs the light, enabling deeper penetration into the tissue [15,22]. According to a recent systematic review [24], most studies have used the wavelength ranging from 640 to 970 nanometers, the power from 0.10 mW to 50 mW, and energy from 3 J/cm^2^ to 90 J/cm^2^. Song JM et al. [25] reported that LLLT is dose-dependent and has tissue repair ability. Furthermore, the use of energy between 6 and 10 J per treatment point and in contact with the tissue surface for a deeper effect is recommended in a study [26].

The biphasic dose-response associated with PBM underscores the importance of using the correct energy density, power, and exposure time to achieve optimal therapeutic effects. As per Arndt’s Law [27], both weak and strong stimuli can influence biological activity, but there is a threshold at which excessive light exposure can suppress cellular function. In this study, by using 200 mW power output, we could achieve increased penetration while minimizing effects such as deep thermal necrosis of the underlying tissue. Additionally, it demonstrated the ability to promote tissue healing and pain relief within the PBM range of 100–500 mW. PBM uses photochemical mechanisms, where the light acts on cells to stimulate mitochondrial enzyme systems to achieve a PBM effect. Since laser outputs are typically low, there are no issues with heat, sound, or vibration [28]. The result of this study demonstrates a comparable clinical outcome of the application of PBM post-endodontic treatment in primary teeth among the 5–9 year age group. The maximum amount of pain reduction is seen at the 4th and 12th h because the action of PBM begins when the laser interacts with the tissue. The studies conducted by Lopes et al. [3], Nunes et al. [12], and Pazin et al. [29] reported a significant pain reduction at the 4th, 6th, 8th, 12th, and 24th h, using 808 nm, 905 nm, and 940 nm diode lasers, respectively. Farhad-Mollashahi et al. [11] and Barciela et al. [30] reported pain reduction at the 24th h and evaluated the pain for a period of 1 week. The laser units used were 970 nm and 660 nm, respectively.

According to Allende et al. [31], the precise biological process by which LLLT produces its analgesic effects implies that it influences inflammation and the production, release, and metabolism of biochemicals that modulate pain at both the central and peripheral levels. Neural conduction block is the result of the LLLT’s induction of neuronal morphological alterations, reduction of mitochondrial membrane potential, and inhibition of rapid axonal transport. According to Parthivi et al. [32], the pain occurred mostly at the 6th and 24th h in the single-visit root canal treatment and significantly decreased after the 24th h. Felician et al. [28] explained that ROS are produced at a low level by normal mitochondrial metabolism; however, in cases where the mitochondrial membrane potential is low due to pre-existing oxidative stress, excitotoxicity, or electron transport inhibition, the production of ROS is reduced. Hence, chronic infections show delayed healing [28]. In our study, PBM targeted both the cervical and periapical areas of deciduous teeth to enhance pain management and healing after dental procedures. These areas are important because of their high nerve innervation and the presence of stem cells capable of promoting tissue repair. By stimulating the alkaline phosphatase activity, increasing collagen and osteocalcin production, and modulating inflammatory responses, PBM not only aids in pain relief but also accelerates tissue healing, ultimately improving the clinical outcomes for pediatric patients undergoing endodontic treatment.

Naseri et al. [33] reported statistically significantly lower pain levels in the laser group (using 808 nm) compared with the placebo group at 4, 8, 24, and 48 h. Çakıcı et al. [1] reported pain reduction after laser application at the 12th and 24th h with a statistically significant difference between groups but no statistical significance at the 4th, 48th, and 72 h.

As pain is a subjective perception, the VAS scale was selected. Extensive evidence in the literature shows that the Visual Analog Scale (VAS) is a simple and efficient tool for measuring subjective experiences, like pain, mood, or discomfort. The VAS offers numerous benefits, including ease and speed, and the numerical scoring (0–100 or 0–10) provides a concrete, quantifiable way to express subjective experiences, making it easier to compare and analyze across individuals or time points even though it is less precise [34].

Although the study was methodologically sound and minimized biases, it had several limitations. One key limitation was the challenge of evaluating post-endodontic pain, as pain measurement is highly subjective. Each individual’s pain threshold is unique, which makes pain assessment inherently variable. In our study, we selected both maxillary and mandibular molars, diagnosed with irreversible pulpitis. The anatomical characteristics of these teeth could have influenced both the presence and intensity of pain. Future studies with larger sample sizes, split-mouth designs, and objective pain assessments could provide stronger evidence for the use of Low-Level Laser Therapy as a reliable and effective method for managing postoperative pain in pediatric endodontics.

## 5. Conclusions

Laser photobiomodulation is a viable, non-pharmacological alternative for managing postoperative pain in children following endodontic procedures on primary teeth. Its ability to reduce discomfort, speed recovery, and enhance patient satisfaction makes it an invaluable tool in pediatric dentistry. As more research clarifies the optimal treatment protocols and long-term benefits, PBM is likely to become an integral part of pain management strategies in pediatric endodontics, improving the overall quality of care and patient experience.

## Figures and Tables

**Table 1:** Comparison of baseline characteristics between groups.

Variables	Control	PBM	*p*
Age in years	6.32 ± 1.84	6.90 ± 1.21	0.772
(Mean ± SD)			
Gender (Male/Female)	8/12	10/10	0.290
VAS scores in	4 (0–3)	4 (0–4)	0.491
Median (Range)			
Tooth treated	4/8	6/7	
Upper/Lower 1st molar			
Upper/Lower 2nd molar	3/5	4/4	0.259

**Table 2:** Comparison of VAS scores across interventional groups.

Time (Hours)	Groups	Median (Range)	Difference Between Groups’ Median (Range)	95% CI	Test Value, *p* Value
4	Control	0 (0–6)	0 (−2–6) *	−1.58	Z = −1.8
	PBM	0 (0–2)		−0.01	*p* = 0.05
12	Control	1.5 (0–4) ^a^	1.5 (−1–4) *	−2.04	Z = −3.32
	PBM	0 (0–1)		−0.65	*p* = 0.001
24	Control	1 (0–4)	0 (−1–3) *	−1.41	Z = −1.9
	PBM	0 (0–2)		−0.08	*p* = 0.05
48	Control	0 (0–1) ^a^	0 (0–1)	−0.23	Z = −1.43
				−0.03	*p* = 0.15

PBM—Laser photobiomoduation group, VAS—Visual Analogue scale score, z—Mann–Whitney U value, *p*—Probability value, ^a^ Intragroup Fredman test *p* value 0.05, * Significant difference at *p* ≤ 0.05, CI—Confidence Interval.

## Data Availability

The data presented in this study are available on request from the corresponding author.
